# Correlation Analysis of Histomorphometry and Motor Neurography in the Median Nerve Rat Model

**Published:** 2014-04-09

**Authors:** Theodora Manoli, Frank Werdin, Hannes Gruessinger, Nektarios Sinis, Jennifer Lynn Schiefer, Patrick Jaminet, Stefano Geuna, Hans-Eberhard Schaller

**Affiliations:** ^a^Clinic of Hand, Plastic, and Reconstructive Surgery with Burn Unit, BG-Trauma Centre, University of Tuebingen, Schnarrenbergstr. 95, 72076 Tuebingen, Germany; ^b^Department of Clinical and Biological Sciences, University of Turin, San Luigi Hospital, Orbassano (TO), Azienda Ospedaliera San Luigi - Regione Gonzole 10, 10043 Turin, Italy

**Keywords:** neurography, electrophysiology, morphometry, nerve regeneration, median nerve

## Abstract

**Objective:** Standard methods to evaluate the functional regeneration after injury of the rat median nerve are insufficient to identify any further differences of axonal nerve regeneration after restitution of motor recovery is completed. An important complementary method for assessing such differences is a histomorphometric analysis of the distal to lesion nerve fibers. Recently, an electrophysiological method has been proposed as a sensitive method to examine the quality of axonal nerve regeneration. **Methods:** A linear regression analysis has been performed to correlate histomorphometric and neurographic data originating from 31 rats subjected to neurotmesis and immediate reconstruction of their right median nerve. **Results:** A significant linear correlation between the velocity of neuromuscular conduction and the total number of nerve fibers (*P* = .037) as well as between the amplitude of compound muscle action potential and the total number of nerve fibers (*P* = .026) has been identified. Interestingly, a significant correlation between the velocity of neuromuscular conduction and the square root of the cross-sectional area of the nerve could be found (*P* = .008). This corresponds to a linear correlation between the velocity of neuromuscular conduction and the radius of the nerve. **Conclusion:** These results contribute in a better interpretation of morphological predictors of nerve regeneration and verify the previously described electrophysiological assessment in the median nerve rat model as a valid method.

The median nerve model of the rat became a popular tool to examine peripheral nerve regeneration under several conditions in the last years. The functional recovery can be simply and reliably assessed by the so-called grasping test as well as by weighing the flexor digitorum sublimis muscle.^1^ In most cases, the grasp force recovers almost completely in about 3 months after nerve lesion. A further and precious supplementary tool to quantify the axonal regeneration is the histomorphometric analysis of the distal to lesion nerve segment.^2^ With this method, parameters such as nerve cross-sectional area, total fiber number, fiber density, diameter of fibers and axons, and myelin thickness can be calculated.

Recently, an electrophysiological method to perform a motor neurography in the median nerve rat model has been established by our group.^3^ With this method, parameters like the threshold to evoke a compound muscle action potential (CMAP), latency, CMAP and velocity of neuromuscular transduction can be assessed by a standardized procedure. The development of motor neurography for the median nerve rat model gave us the opportunity to get more information about the quality and extent of the axonal regeneration that has taken place over several time points.

The purpose of the actual study was to correlate electrophysiological parameters and histomorphological findings from the median nerve model of the rat, after functional recovery was completed. Furthermore, our results could validate both methods as more sensible tools to evaluate axonal regeneration, compared to the standard functional tests.

## MATERIALS AND METHODS

### Data acquisition

The electrophysiological and histomorphological data used for our correlation analysis originated from 2 already published works of our department.^4^^,^^5^ In both works, the median nerve model of Wistar adult female rats, weighing 220 to 250 g each, was used. In total, 31 rats originating from these works were subjected to both electrophysiological and histomorphological analysis 12 weeks after surgery. Experiments have been carried out in accordance to EC Directive 86/609/EEC for animal experiments.

The distribution of animals according to their treatment after neurotmesis of the right median nerve is depicted in Table 1. The electrophysiological parameters assessed by our standard protocol^3^ and used for the actual correlation analysis have been as follows:

– The threshold [V] of stimulus to provoke a CMAP
– The transduction velocity or v [m/s], which was calculated by the distance between the stimulus and the electrode placed in the flexor sublimis muscle divided by the latency between the stimulus and the beginning of the CMAP, and
– the amplitude of the CMAP [μV]

The histomorphometric data assessment^2^ used for the actual study included the following parameters:

– The cross-sectional area of the nerve
– The total fiber number (N)
– Fiber density
– Fiber diameter (D)
– Axon diameter (d)
– Myelin thickness (M), and
– Axon/fiber ratio or g ratio (g = d/D)

### Statistical analysis

Data analysis was performed using version 2.11.0 of R software and its package “stats” to correlate electrophysiological and histomorphological parameters.^6^ Since the histomorphological parameters can be considered as independent and the electrophysiological parameters as dependent variables, linear regression analysis has been chosen to correlate the 2 methods. A normal distribution was expected for all parameters. Linear regression analyses have been performed between 1 of the 3 electrophysiological parameters and 1 of the 7 different histomorphological parameters mentioned earlier at a time. The algorithm used to fit linear models was the one proposed by Chambers.^7^ The level of significance after applying an *F* statistic was set by a *P* < .05. The Institute of Biometry of the University of Tuebingen has validated the statistical analysis. The work described in the actual article fulfils the Uniform Requirements for manuscripts submitted to Biomedical journals.

## RESULTS

**Figure 1:**
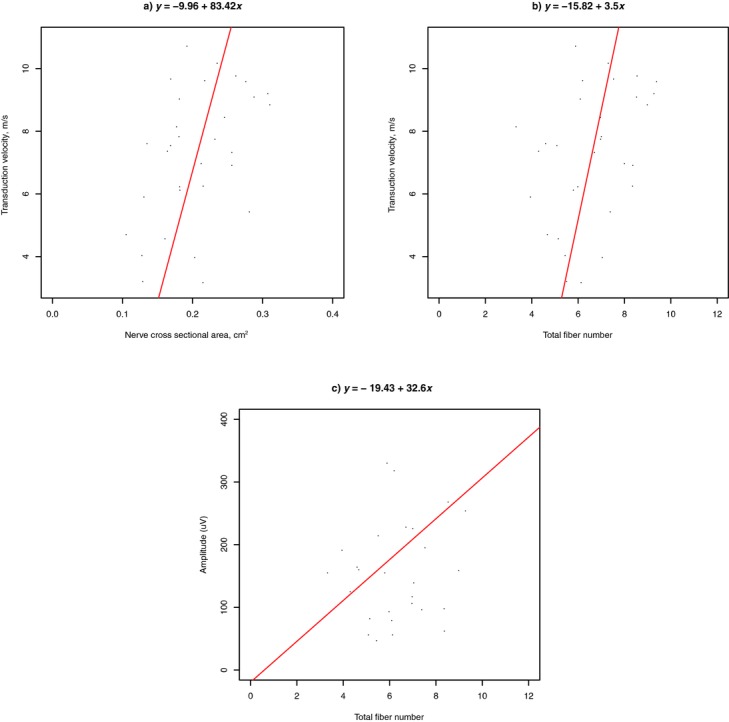
Fitted linear models (red lines) after regression analysis; between (*a*) v and cross-sectional area of the nerve, (*b*) v and N, and (*c*) amplitude and N.

**Figure 2:**
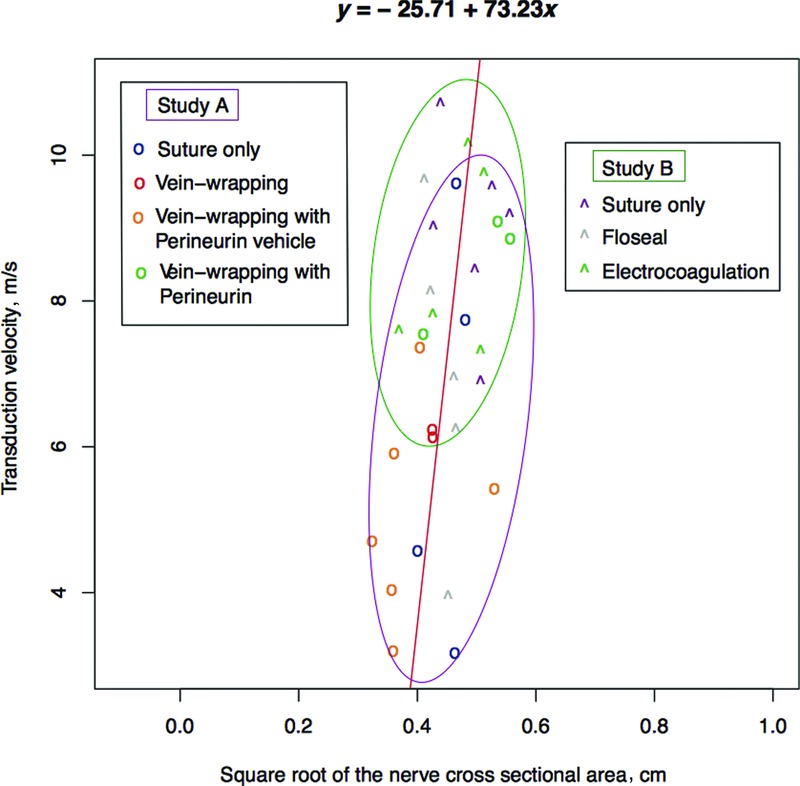
Fitted linear model (red line, *y* = −25.71 + 73.23*x*) after regression analysis between the square root of the cross-sectional area of the nerve and the transduction velocity. Data originating from study A are depicted by “o” and data originating from study B by “ˆ.” The different colors present the different treatments, as described in the legends. Magenta ellipse includes all measurements originating from study A, while green ellipse includes all measurements but one originating from study B.

The *P* values obtained by the *F* statistic applied to find significant correlations between the histomorphometric and neurographic data after performing a linear regression analysis are shown in Table 2. Significant linear correlations with *P* < .05 could be found in 3 cases. These were between (*a*) transduction velocity and cross-sectional area of the nerve (*y* = −9.96 + 83.42*x*, *P* = .009), (*b*) transduction velocity and total fiber number (*y* = −15.82 + 83.42*x*, *P* = .037), and (*c*) amplitude and total fiber number (*y* = –19.43 + 32.6*x*, *P* = .026). These 3 linear models are depicted in Figures 1a-c. Concerning the first case, an even more significant linear correlation could be observed between the square root of the cross-sectional area of the nerve and the transduction velocity (*y* = –25.71 + 73.23*x*, *P* = .008). Having in mind the formula for calculation of the circle area, A = π*r*^2^, it can be concluded that transduction velocity is linear correlated to the nerve radius or diameter. Figure 2 is a graphical presentation of this linear fitted model (red line). Data originating from the 2 different studies are presented with different symbols and different colors according to their treatment, as described in the legends. Such graphs may be useful tools to compare different treatments or different studies. In this case, we can conclude that animals of study B achieved a higher mean transduction velocity than the animals of study A. This is probably due to the generally slightly larger diameter of the median nerves of the rats used for study B with a mean value of 0.217 μm^2^ than for study A with a mean value of 0.198 μm^2^.

## DISCUSSION

In this study, a good linear correlation could be obtained between 2 neurographic parameters (transduction velocity and amplitude) and the total fiber number obtained by the histomorphometric analysis. Moreover, a very good linear correlation was obtained between transduction velocity and nerve diameter. Our results illustrate the progress in the improvement of histomorphometry and especially of neurography in rodents, as no significant correlations between these methods could be obtained in the early phase after completion of functional recovery in several previous studies.^8^^-^10 In a previous study, a moderate to good correlation between amplitude and fiber counts demonstrating a diameter 3 to 5 μm in the peroneal nerve of the rabbit, 4 to 15 weeks after repair, could be obtained.^11^

Dellon and Mackinnon first demonstrated a strong positive correlation between conduction velocity and fiber diameter as well as between amplitude and number of nerve fibers 1 year after nerve repair.^12^ In our study, no significant correlation between the conduction velocity and fiber or axon diameter could be obtained. This is probably due to the early time point (12 weeks) of neurographic and histomorphometric assessment. Previous studies evaluating nerve regeneration in the rat across a nerve repair demonstrated that the total number of fibers that reached the distal segment varied with time. In the first few months after surgery, the number of axons will increase dramatically due to axonal sprouting. Some of the axon sprouts make appropriate distal connections and some of them do not, which results in a later decrease in fibers. The number of axons reach a plateau between 6 and 9 months after repair and return to normal levels within 1 year in the rat model.^13^^,^^14^ Thus, changes in the number of nerve fibers are expected in the first year after nerve repair. Moreover, most authors found a decrease in axon diameter concurring with a smaller decrease in fibre diameter up 1.5 years after nerve repair.

It has been shown that the ratio of axon to fiber diameter (d/D) or g-ratio remains quite constant during regeneration and that conduction velocity depends on a small number of the largest axons in the nerve.^15^ However, no significant linear correlation between conduction velocity and the maximal g-ratio could be found in our data. A significant correlation between myelin thickness and conduction velocity was expected^16^^,^^17^ but could not be verified in our data. An explanation could be that a significant part of myelinated fibers may have been sensory fibers that do not have an impact in motor neurography. However, the good correlation between transduction velocity and total fiber number implies that the total fiber number may be a better morphological predictor of an effective peripheral nerve regeneration of a mixed nerve than the myelin thickness, especially during the early phase of regeneration.

## CONCLUSIONS

Interesting correlations of histomorphometric and neurographic data originating from 2 studies using the median nerve rat model could be observed. Our results could validate motor neurography as a sensible method for the assessment of regeneration in the median nerve model of the rat. These findings are also important since histomorphometric and electrophysiologic measurements enable a more subtle interpretation of peripheral nerve regeneration quality than functional tests do and do not always correlate with nerve sensory or motor function.^18^^,^^19^ A combined analysis of histomorphometry and motor neurography enables an even more precise evaluation of the axonal regeneration in the median nerve model of the rat, making it a powerful model to investigate several conditions that may influence peripheral nerve regeneration, or new reconstruction methods and strategies before applying them on a clinical level.

## Figures and Tables

**Table 1:** Distribution of animals according to their surgical treatment as described in studies A^4^ and B^5^

Study	Treatment	Number of animals
A	Direct suture	3
A	Direct suture plus vein-graft wrapping	4
A	Direct suture plus vein-graft wrapping filled with Perineurin vehicle	2
A	Direct suture plus vein-graft wrapping filled with Perineurin	6
B	Direct suture	5
B	FloSeal application to the nerve stumps and direct suture	6
B	Electrocoagulation of the nerve stumps and direct suture	5

**Table 2:** F-statistic of the binary regression analyses between the histomorphometric and neurographic data

	Neurographic parameters		
	Threshold	Transduction velocity	Amplitude
**Histomorphometric parameters**			
**Cross sectional area**	0.562	**0.009**	0.083
**Total fiber number (N)**	0.201	**0.037**	**0.026**
**Fiber density**	0.406	0.133	0.869
**Fiber diameter (D)**	0.543	0.749	0.556
**Axon diameter (d)**	0.603	0.680	0.767
**Myelin thickness (M)**	0.860	0.859	0.514
**G ratio (d/D)**	0.892	0.689	0.813

The numbers correspond to *P* values. Significant values (*P* < .05) are marked in grey.
